# Supplementary material for: Prenatal and postnatal challenges affect the hypothalamic molecular pathways that regulate hormonal levels
Source: PLoS One. 2023 Oct 18;18(10):e0292952. doi: 10.1371/journal.pone.0292952 (PMC10584192; doi:10.1371/journal.pone.0292952)
Supplement: Attachment — Submitted filename: PONE-D-23-19245Hypo22ReplytorTviewersPLOSone_sep072023.docx [file pone.0292952.s007.docx]

September 7, 2023

Reply to the editorial and reviewers’ recommendations on the manuscript PONE-D-23-19245

Dear Professor Dr. Hrncic and reviewer,

We appreciate for the opportunity to revise our manuscript PONE-D-23-19245. We have modified the submission to address questions and integrate the recommendations offered to us. The suggestions have strengthened our manuscript, and we thank the reviewers for their feedback.

Below is the list of recommendations made by the editorial team and reviewer, and in our point-by-point reply, we describe the manuscript modifications that address the suggestions.

Thank you

Sandra Rodriguez Zas

Journal Requirements:

When submitting your revision, we need you to address these additional requirements.

1. Please ensure that your manuscript meets PLOS ONE's style requirements, including those for file naming. The PLOS ONE style templates can be found at

https://journals.plos.org/plosone/s/file?id=wjVg/PLOSOne_formatting_sample_main_body.pdf and

<https://journals.plos.org/plosone/s/file?id=ba62/PLOSOne_formatting_sample_title_authors_affiliations.pdf>

REPLY. Agree. We have revised the manuscript to meet the PLOS ONE’s style requirements listed in both links and corresponding changes are highlighted.

2. Please expand the acronym “USDA, NIFA, AFRI, ILLU” (as indicated in your financial disclosure) so that it states the name of your funders in full. This information should be included in your cover letter; we will change the online submission form on your behalf.

REPLY. Agree. We have expanded the acronyms in the manuscript and in the financial disclosure. United States Department of Agriculture (USDA), National Institute of Food and Agriculture (NIFA), Agriculture and Food Research Initiative (AFR)I, University of Illinois at Urbana-Champaign Experiment Station (ILLU)

3. Thank you for stating the following financial disclosure:

"This research was funded by USDA NIFA AFRI award number 2018-67015-27413, USDA NIFA ILLU award number 2022-38420-38610, and NIH NIDA award number P30 DA018310."

Please state what role the funders took in the study. If the funders had no role, please state: "The funders had no role in study design, data collection and analysis, decision to publish, or preparation of the manuscript." If this statement is not correct you must amend it as needed.

Please include this amended Role of Funder statement in your cover letter; we will change the online submission form on your behalf.

REPLY. Agree. We have included the amended Role of Funder statement in the cover page. Thank you for changing the online submission form on our behalf.

4. In your Data Availability statement, you have not specified where the minimal data set underlying the results described in your manuscript can be found. PLOS defines a study's minimal data set as the underlying data used to reach the conclusions drawn in the manuscript and any additional data required to replicate the reported study findings in their entirety. All PLOS journals require that the minimal data set be made fully available. For more information about our data policy, please see http://journals.plos.org/plosone/s/data-availability.

REPLY. Agree. The Data Availability statement now includes the location of the minimal data set underlying the results. Accession number is GSE227725.

Upon re-submitting your revised manuscript, please upload your study’s minimal underlying data set as either Supporting Information files or to a stable, public repository and include the relevant URLs, DOIs, or accession numbers within your revised cover letter. For a list of acceptable repositories, please see http://journals.plos.org/plosone/s/data-availability#loc-recommended-repositories. Any potentially identifying patient information must be fully anonymized.

We will update your Data Availability statement to reflect the information you provide in your cover letter.

REPLY. Agree. We have added the location of the study’s minimal underlying data set in the revised cover letter. Thank you for updating the Data Availability statement on your behalf.

5. We note that you have stated that you will provide repository information for your data at acceptance. Should your manuscript be accepted for publication, we will hold it until you provide the relevant accession numbers or DOIs necessary to access your data. If you wish to make changes to your Data Availability statement, please describe these changes in your cover letter and we will update your Data Availability statement to reflect the information you provide.

REPLY. Agree. The repository information for our data has been provided in the manuscript and in the cover letter. Accession number is GSE227725. The access token for the editorial team and reviewers was provided in the cover letter and submission form. Unrestricted public access to the data will be available at the time of the journal’s acceptance.

Reply to reviewer

Reviewer #1: This work used RNA sequencing to study how prenatal challenge with maternal immune activation (MIA) and postnatal challenge with nursing impact the molecular pathways of hypothalamic pathways, and examine the effects are different among female and male pigs. The main concerns are as follows,

1) Instead of whole-transcriptome sequencing, the work used a target panel of 222 genes. Why? How these genes were chosen? How this limitation will affect the functional analysis of differentially expressed genes (DEGs)?

REPLY. Agree. Clarifications were added in page 6 and in page 15. A whole-transcriptome analysis was undertaken. Please refer to page 5, second paragraph about the “test the effects of prenatal inflammation, nursing withdrawal, sex, and interactions on 18,295 genes”. The reference to 222 genes in the Discussion correspond to the number of genes that presented differential expression at FDR-adjusted p value < 0.05 (page 15 first paragraph).

2) The main result is to present the analysis results of DEGs across three factors, functional significance and the possible regulation mechanism. The current form is hard to follow. The authors may consider to reorganize the results in a more intuitive way.

REPLY. We agree in that the flow of result presentation must be amenable to readers while meeting scientific standards. The order of result presentation follows statistical and biological better practices. Following the logical approach of deductive reasoning, the progress of the result presentation goes from functional categories, corresponding genes and interactions impacted by the factors studied (Tables 1 and 2 and Figs 2-3). Subsequently, the presentation of results focuses up-regulated or down-regulated functional categories (Table 4) and ends with transcription factors. Also consistent with better practices, results within a table were obtained from a common model, method, and specifications.

3) Main result is focused on the hormonal regulation pathway. However, a lot of discussion is on the olfactory transduction pathway while only one enrichment result was presented about the pathway.

REPLY. Agree. Clarifications were added in page 10, and 18. The network of genes in the hormonal regulation pathway was selected for visualization across 12 contrasts because of the significant enrichment, and because it includes genes from other enriched pathways including regulation of transcription by RNA polymerase II, G-protein coupled receptor signaling pathway, cellular response to endogenous stimulus, regulation of signaling receptor activity. Also, the number of genes annotated to the hormonal regulatory pathway is amenable to interpretable visualization. The larger number of genes annotated to the olfactory transduction pathway resulted in a denser network that challenged accessible visualization and interpretation for the 12 group comparisons reported.

4) There is no confirmation from the protein levels on the findings.

REPLY. Agree. A clarification was added in page 6. We agree with the implementation of strategies to minimize false positive results, and in the unbiased validation of results whenever possible. The large number of samples analyzed (72 individuals) and significant genes across effects and contrasts prevents an unbiased confirmation of all group contrasts and 220 genes discussed using protein-based analysis. The replicability of the findings is supported by the conservative criteria in the analysis, and genes and categories selected for interpretation (i.e., simultaneous FDR adjusted p-value < 0.05). Also, genes with low sequence reads in any one group were not reported (Materials and methods section). To convey high degree of robustness in the findings, our discussion highlighted differentially expressed genes that were both supported by enriched functional categories and have been previously associated with maternal immune activation, stress, and sex differences as evidenced by our thorough reference list.

Minor points:

1) Figure 1 is hard to digest. Only the DEGs are included and the box-whisker plot seems inappropriate since DEGs are either upregulated (logFC >0) or downregulated (logFC<0). Non-DEGs should have a logFC close to 0.

REPLY. Agree. A clarification was added in page 6. We agree that have Fig 1 depicted all 18,295 genes analyzed, the box-whisker plots would have been centered around 0. Fig 1 focuses on the differentially expressed genes and therefore the plots will not necessarily be centered around 0. The plots aim at highlighting that the effects studied do not have a balanced effect in up-regulating some genes and down-regulating in similar magnitude or quantity.

2) How are the expression levels of 222 genes quantified? Esp in the comparisons between Ctrl and MIA, and between Wea and Nur, the logFCs of DEGs were biased towards the negative side and the positive side, respectively?

REPLY. Agree. Clarifications were added in pages 5 and 6. We agree that the description of the quantification is important in assessing the findings. A generalized linear model was used to test the effects of prenatal inflammation, nursing withdrawal, sex, and interactions on 18,295 genes analysis. Among these, some genes were differentially expressed at FDR-adjusted p value < 0.05 between MIA and Ctrl and others were differentially expressed between Wea and Nur. Fig 1 demonstrates that most of the impacted genes were over-expressed in individuals exposed to MIA and in individuals exposed to stress.

3) “Sex” is a different factor from other two. “prenatal challenge with sex” and other similar descriptions are confusing and misleading. The authors may consider rewrite the description on the sex factor.

REPLY. Agree. Clarifications were added in the abstract and in page 8. We agree that while the prenatal and postnatal challenges are distinct in nature to sex differences. Following standard statistical approaches, we tested the effect of each one of the factors (maternal immune activation, stress, and sex) and also their interaction. These interactions aim at identifying whether the effect of maternal immune activation (or stress) depends or varies with sex. In consideration that 79 genes presented significant interaction of either prenatal or postnatal challenge with sex, we favor reporting these interactions in paragraphs proximal to interpretations that focus on the challenges to remind readers of the sex-dependent nature of some of the profiles detected.
